# Household income is associated with attack frequency, but not with the prevalence of headache: an analysis of self-reported headache in the general population in Germany

**DOI:** 10.1186/s10194-024-01844-w

**Published:** 2024-10-01

**Authors:** Britta Müller, Charly Gaul, Olaf Reis, Tim P. Jürgens, Peter Kropp, Ruth Ruscheweyh, Andreas Straube, Elmar Brähler, Stefanie Förderreuther, Florian Rimmele, Thomas Dresler

**Affiliations:** 1 Institute of Medical Psychology and Medical Sociology, Rostock University Medical Center, Gehlsheimer Str. 20, Rostock, 18147 Germany; 2 Headache Center Frankfurt, Frankfurt, Germany; 3 Department of Child and Adolescent Psychiatry and Neurology, University Medical Center Rostock, Rostock, Germany; 4 German Center for Child and Adolescent Health (DZKJ), partner site Greifswald/ Rostock, Rostock, Germany; 5 Department of Neurology, University Medical Center Rostock, Rostock, Germany; 6 Department of Neurology, LMU University Hospital, LMU Munich, Munich, Germany; 7 Integrated Research and Treatment Center (IFB) Adiposity Diseases - Behavioral Medicine, Psychosomatic Medicine and Psychotherapy, University of Leipzig Medical Center, Leipzig, Germany; 8 Department of Psychosomatic Medicine and Psychotherapy, University Medical Center of the Johannes Gutenberg-University, Mainz, Germany; 9 Department of Neurology, Headache Center North-East, University Medical Center Rostock, Rostock, Germany; 10 Department of Psychiatry and Psychotherapy, Tübingen Center for Mental Health, University Hospital Tübingen, Tübingen, Germany; 11 LEAD Graduate School & Research Network, University of Tübingen, Tübingen, Germany; 12 German Center for Mental Health (DZPG), Partner Site Tübingen, Tübingen, Germany

**Keywords:** Chronic headache, Education, Epiosodic headache, Headache prevalence, Headache frequency, Household income, Socioeconomic position, Net household income

## Abstract

**Background:**

Headache disorders are among the most prevalent neurological disorders worldwide. However, whether groups differing in socioeconomic position (SEP) are disproportionately affected by headache disorders has not yet been adequately clarified. Our aim was to analyse (1) the headache prevalence by socioeconomic position (SEP) and (2) the attack frequency by SEP in a German population-based adult sample.

**Methods:**

Cross-sectional data from a random general population were used. The sample included *N* = 2,189 participants aged ≥ 18 years. SEP was measured using net equivalised income (NEI) and education. A binary logistic regression model tested the effect of SEP in predicting the prevalence of headache in general. Ordinal logistic regressions were modeled to predict the effect of SEP on the likelihood of attack frequency. Attack frequency was categorized in low frequency episodic headache (LFEH: 0–3 days per month), moderate frequency episodic headache (MFEH: 4–14 days per month) and chronic headache (CH: ≥ 15 days per month).

**Results:**

Of the 2,189 participants, 891 reported headache in the last six months. Neither income nor education was associated with headache prevalence. However, significant differences between income groups were found for attack frequency. Compared to participants with NEI > 150%, those with NEI < 60% were 5.21 times more likely (95%CI 2.03, 13.36) to experience higher headache frequency, and those with NEI between 60 and 150% were 2.29 times more likely (95%CI 1.02, 5.11), with adjustments made for a set of potential confounders, including depressive symptoms.

**Conclusions:**

To reduce headache attacks, it is essential to address both low- and middle-income groups affected by headaches. Universal public health prevention campaigns are particularly appropriate.

## Introduction

From a public health perspective, headache disorders, mostly migraine and tension-type headache (TTH), impose a major societal challenge as they are one of the most disabling disorders. According to the Global Burden of Disease (GBD) Study 2019, headache disorders are responsible for 5.4% of total years lived with disability (YLDs) worldwide [1]. Two key parameters for estimating YLDs are *headache prevalence* and *attack frequency* [2]. For targeted use of public-health resources to reduce the burden, it is important to find out which of these parameters is more susceptible to influences. From the public health perspective, it is worth investigating this question with regard to the socioeconomic position (SEP). Worldwide, the SEP has a strong influence on health outcomes: the lower the individual’s SEP, the higher their risk of several diseases and more severe symptoms [3, 4]. However, whether lower SEP groups are also disproportionately affected by headache disorders, measured by prevalence and attack frequency, has not yet been sufficiently clarified. To date, research has focused on analysing headache prevalence, while analyses of headache attacks have been scarce.

**Fig. 1:**
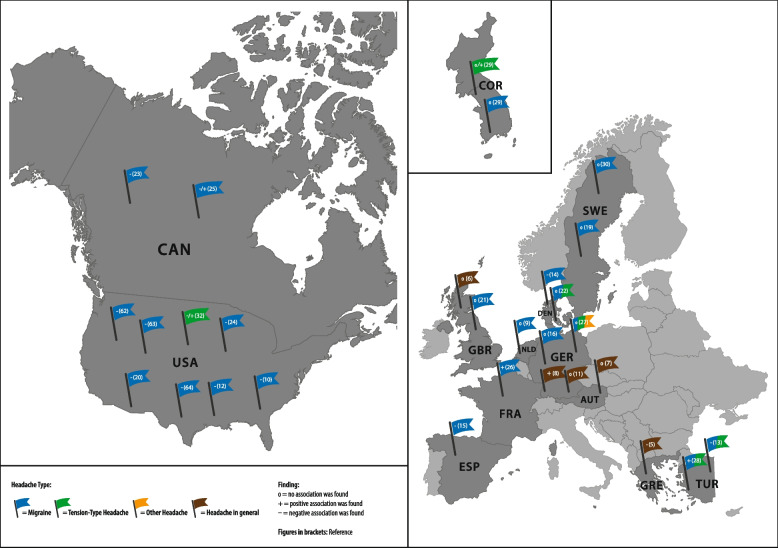
Studies examining the association between headache prevalence and socioeconomic position (focus on OECD countries)

Although there is a large number of studies on the prevalence by SEP, the findings are inconsistent for headache in general [5–8] as well as for migraine [9–30] and TTH [11, 22, 27–29, 31, 32] (see Fig. 1). In addition to different welfare state contexts, different measures for SEP (e.g. education, occupation, and/or income), as well as different sets of control variables may have resulted in heterogeneous results. Although the SEP measures are related, they are not interchangeable. Available literature therefore suggests using multiple socioeconomic indicators rather than a single variable to analyse health by SEP [33, 34]. With regard to control variables, it is noticeable that only one study examined the association between SEP and headache adjusted for depression [15]. This is unfortunate, because both headache [35] and SEP are associated with depression [36].

In contrast to headache prevalence, the association between SEP and attack frequency has been scarcely researched. Particularly for episodic headache (EH) (< 15 headache days per month), which affects most people with headache, while chronic headache (CH) (≥ 15 headache days per month) affects only 3–4% of the population [35–37]. To the best of our knowledge, only three studies analysed the association between EH and SEP. Stewart et al. (2013) found that women and men in the lowest income group were less likely to have ≤ 3 migraine days per month [12]. In the Norwegian HUNT study it was found that low SEP at baseline was associated with increased risk of having 6-14 headache days per month and chronic headache 11 years later, whereas no SEP-specific risks of having 1–6 headache days were found [37]. The authors of the American AMPP study reported a higher likelihood of low SEP in those with 8–14 migraine days per month compared to those with less frequent headache days [38]. However, it is questionable whether this association was maintained when controlling for depressive symptoms, as the literature suggests that depressive symptoms increase with increasing attack frequency [39].

To summarize the current research on headache prevalence and attack frequency across different SEP groups, we examined whether these factors are associated with net eqivalised income (NEI) and education in a German cross-sectional population-based adult sample. These analyses controlled for a range of potentially confounding factors, including depressive symptoms. In this way, the study contributes to a better understanding of the social patterns of headache burden in the population.

## Methods

### Participants

The analysis is based on cross-sectional data from a random general population sample (*N* = 2,510), collected in 2016 in Germany among inhabitants aged 14 years and older [40]. For sample selection, random multi-stage sampling procedures were employed. First, 258 regional sample points in Germany were determined (stage 1). Subsequently, 19 households per sample point were selected using the random-route procedure (stage 2). Members of households, who met the inclusion criteria (age above 14, able to read, and understand German) were randomly selected using the Kish-selection-grid technique (stage 3). 

In total, 4,838 subjects were selected for the study, and 2,514 people participated. All participants provided their written informed consent. Four interviews were not analyzable, resulting in an final data set of 2,510 interviews. Reasons for non-participation included refusal to take part (*n* = 1,453), three unsuccessful attempts to contact the household member (*n* = 863), and illness or incapacity of the selected subjects to follow the interview (*n* = 8). To mitigate selection bias, an adjustment factor was calculated based on the German population structure regarding age, sex, household size, and population by federal state. German population parameters were obtained from the 2016 Microcensus conducted by the German Federal Statistical Office. Using this adjustment factor, a weighted random sample was created that corresponded to the structure of the German population with regard to these factors [41]. Detailed information on the adjustment weighting can be obtained from the first author.

**Fig. 2:**
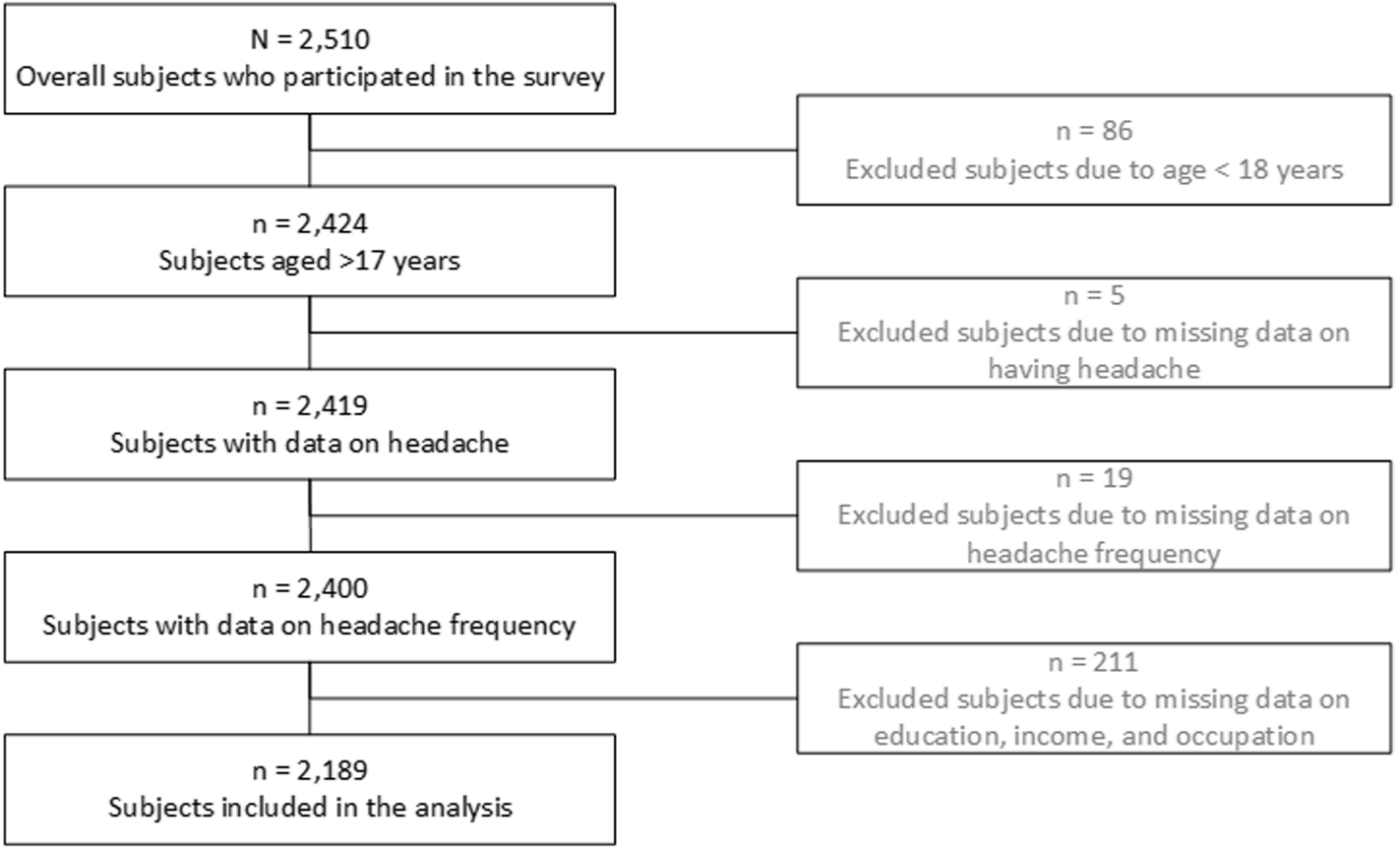
Flowchart for inclusion and exclusion criteria

For the present analysis, only adults were included, resulting in the exclusion of 86 individuals due to age < 18 years. The decision to exclude data from those under 18 was based on the mitigated validity of their reports: adolescents’ reports of their parents’ income generally show high levels of missing or invalid data [42, 43]. Additionally, adolescents between the ages of 14 and 18 are likely to be in school, meaning they do not yet have an educational qualification, and therefore SEP based on education can only be estimated inaccurately. Furthermore, 235 individuals were excluded due to missing data on headache prevalence, headache frequency or SEP, resulting in a final sample of *N* = 2,189 participants (see Fig. 2).

### Questionnaire

A standardized questionnaire was used to collect data on headache, including its treatment and relevant sociodemographic variables [44]. The sociodemographic data were collected using face-to-face interviews. The questionnaire section on headache and its treatment was filled in by the respondents themselves.

#### Dependent variables

##### Headache prevalence

Information on headache prevalence is based on the participants' statements on whether they had experienced headaches in the last six months.

##### Headache frequency

Headache frequency was assessed using a five-point ordinal scale: (1) < 1 day per month; (2) 1–3 days per month; (3) 4–14 days per month; (4) > 14 days per month but not daily; (5) and daily. For statistical analysis, the five categories were converted into three categories. The first category included headaches that occur less frequently than four times a month, named “Low Frequency Episodic Headache” (LFEH). The second category included headaches occurring 4–14 days per month, labelled as “Moderate Frequency Episodic Headache” (MFEH). The third category included headaches that occur at least 15 days a month, labelled as “Chronic Headache” (CH). Individuals without headache were assigned to the frequency category “No headache”.

#### Independent variables

Income and education were used as proxies for the SEP. NEI was calculated from the data on household size and monthly net household income by dividing the monthly net household income by the square root of the number of persons living in the household [45]. Based on the NEI the sample was split into three relative income position categories. The lowest category covers all participants with an NEI below 60% of median income of the sample [46]. The second category ranges from 60 to 150% of median income. The third category covers participants with an NEI more than 150% of median income. Education was summarized according the International Standard Classification of Education 97 (ISCED-97) into three classes: lower school education (ISCED-level 1/2), intermediate school education (ISCED-level 3/4), and higher school education (ISCED-level 5/6) [47].

#### Covariates

Employment status was originally assessed with nine categories which were summarized for the analyse into three groups: employees; non-employed; unemployed. Sociodemographic variables comprised sex, age, marital status, living with partner, minor children living in participant's household. The residential environment was classified into rural and urban areas based on the sampling plan. A rural region was defined as less than 20,000 inhabitants living in a community that was neither close to large cities nor part of a city-region or metropolitan area [48]. Self-reported data on body weight and height were collected to calculate the Body Mass Index (BMI) (kg/m^2^). Obesity was defined as a BMI > 30 kg/m^2^ and was used as a dichotomous variable (“obesity yes/no”) [49]. Depressive symptoms were measured with the subscale of the Patient Health Questionnaire (PHQ-4) that encompasses two items and has sum scores ranging from 0 to 6. Scores ≥ 3 indicate the presence of significant depressive symptoms. The scales showed acceptable reliability with McDonald's omega of ω = 0.85 for PHQ-4 [50]. Depressive symptoms were used as a dichotomous variable (“depressive symptoms yes/no”). Use of outpatient care was measured by asking those participants who reported that they had headaches in the last 6 months whether they had ever consulted a physician (or more than one) for headache (yes/no).

### Statistical analysis

The sample structure was compared to the population structure regarding a representative distribution by household size, age, sex, and federal state. To correct for deviations of the sample, a weighting factor was applied to improve the representativeness of the sample. All analyses were conducted with the weighted sample; however, absolute numbers of cases are presented unweighted, as the sum of weighted factors differs from that of unweighted factors in a subsample analysis.

Pearson’s χ^2^ Test was used for bivariate analysis. The interpretation of results between categorical variables was based on the recommendations by Agresti [51]. This author suggests the use of adjusted standardized residuals to evaluate deviations between observed and expected frequencies. An adjusted residual exceeding 2 or 3 in absolute value indicates a rather unlikely deviation which can be interpreted as significant. In the present analysis, deviations exceeding a value of 2 were considered significant.

A binary logistic regression was performed to predict the effect of SEP, adjusted for sociodemographics, family and health-related characteristics, on the likelihood of having had any headache in the last 6 months. All variables were simultaneously entered as predictors in the equation.

Ordinal logistic regressions were modeled to predict the effect of SEP on the likelihood of headache attack frequency as ordinal dependent variable (“LFEH”, “MFEH”, and “CH”). We excluded individuals without headaches from this regression analysis to better identify particularly vulnerable subgroups among those affected. These analyses were sequentially adjusted for a set of sociodemographic and family variables (Model 2), and health-related variables (Model 3). Prerequisites of ordinal logistic regressions were tested. There were no violations of the assumption of no multicollinearity and proportional odds. The model fit was assessed using four indices: 1) *Likelihood Ratio Test* (compares the null deviance to the residual deviance; a large difference suggests the model explains data variability well; the difference follows a χ^2^ distribution, and a significant *p*-value indicates that the model with predictors is significantly better than the null model), 2) *Goodness-of-Fit Tests* (includes Pearson’s χ^2^ Test, which compares observed and expected frequencies, and the Deviance Goodness-of-Fit Test, which compares observed outcomes to model predictions; non-significant *p*-values suggest a good model fit), 3) *Pseudo-R*^*2*^ *Measure* (indicates the proportion of variability explained by the model; common measures include Nagelkerke's *R*^2^, which adjusts Cox and Snell's *R*^2^ to reach 1; higher values indicate a better fit), and 4) *Test of Proportional Odds* (assesses whether the relationship between each pair of outcome groups is consistent; non-significant *p*-values suggest that the proportional odds assumption holds and the model is appropriate).

The odds ratio (*OR*) and 95% confidence intervals (*CI*) were computed using the formulas outlined below:

$$OR = exp (b)$$

$$95\%CI=\text{exp}\left(\text{ln}\left(OR\right)\pm 1.96\cdot SE\left\{\text{ln}\left(OR\right)\right\}\right)$$

A *p* value < 0.05 was considered statistically significant. Statistical analyses were performed using IBM SPSS Statistics 27 (SPSS Inc., Chicago, IL, USA).

## Results

### Sample characteristics

**Fig. 3:**
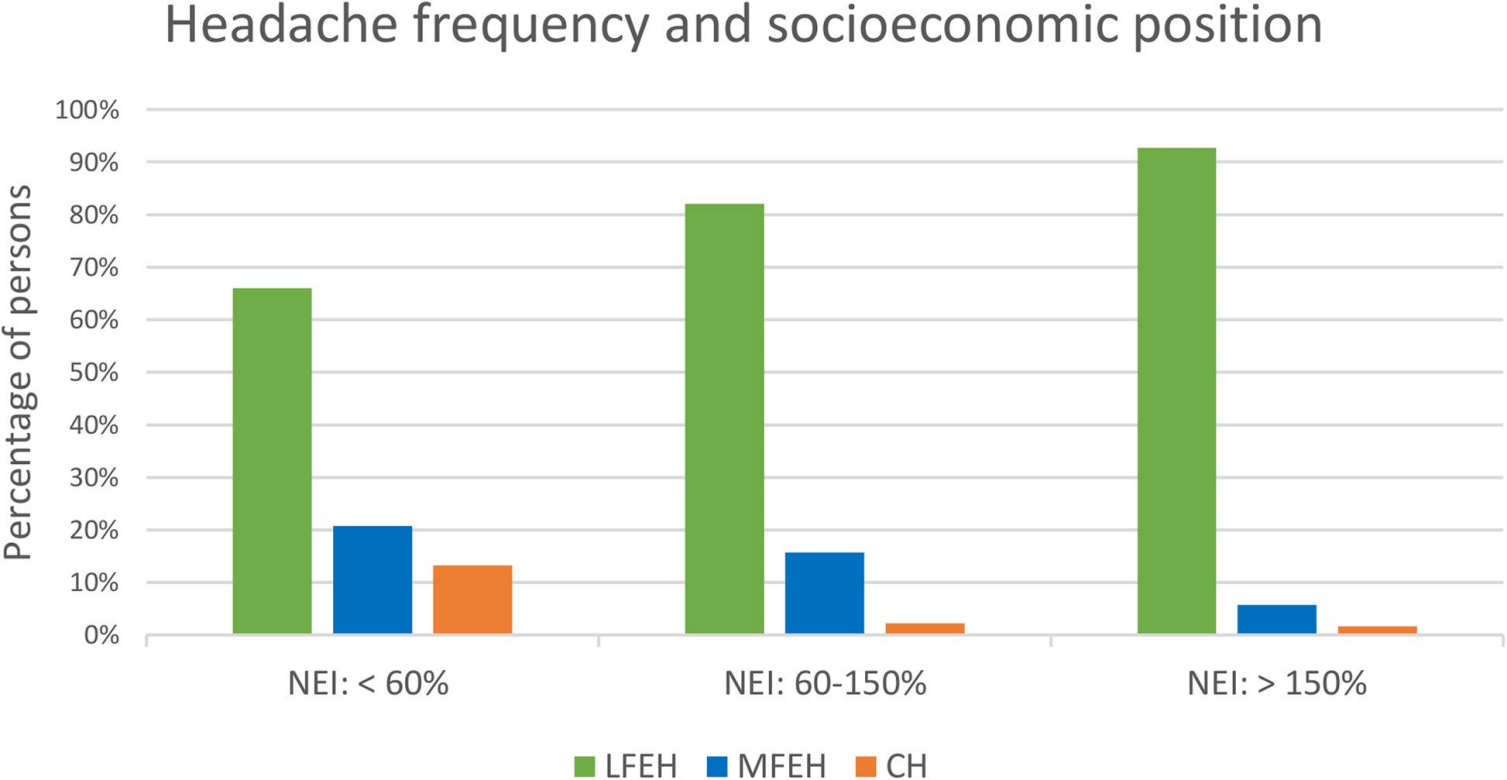
Frequency of headache as a function of SEP (weighted random sample). Legend: SEP, socioeconomic position, LFEH, low frequency episodic headache (< 4 days per month); MFEH, medium frequency episodic headache (4–14 days per month); CH, chronic headache (≥ 15 days per month)

The sample characteristics regarding headache, sociodemographic and health-related variables are shown in Table 1. The weighted 6-month prevalence was 40.2% of all types of headache (95% *CI*: 38.1%-42.3%). LFEH affected 32.8% (95% *CI*: 30.8%-34.8%) of the participants. 6.0% (95% *CI*: 5.0%-7.0%) reported MFEH. CH affected 1.5% of the participants (95% *CI*: 1.0%-2.0%). NEI and education correlated weakly, Spearman’s ρ = 0.231, *p* < 0.001. Regarding the bivariate analysis, there was an association between NEI and headache frequency, χ^2^(4) = 47.6, *p* < 0.001. Participants with an NEI of less than 60% of the median income were over-represented among those with CH. Participants with more than 150% of the median income were over-represented in the group with LFEH (see Fig. 3). No association between education and headache frequency was found, χ^2^(4) = 4.2, *p* = 0.375.

**Table 1:** Descriptive statistics of indicators used in the analysis:Columns 3–7: % (CI) (weighted dataset); Column 8: absolute frequencies (unweighted dataset), % (CI) (weighted dataset)

Variable	Category % (95%-CI)	Participants without headache	Participants with headache				Total	
		**(** ***n*** ** = 1,298)**	**LFEH (** ***n*** ** = 725)**	**MFEH (** ***n*** ** = 133)**	**CH (** ***n*** ** = 33)**	**All (** ***n*** ** = 891)**	**(** ***N*** ** = 2,189)**	
Net equivalised income (NEI)	< 60%	10.7 (9.0, 12.4)	10.0 (7.8, 12.2)	17.2 (10.7, 23.7)	46.7 (28.9, 64.6)	12.3 (10.1, 14.5)	*n* = 284; 11.3 (8.4, 10.8)	
Missing: 0	60–150%	75.4 (73.0, 77.8)	73.8 (70.6, 77.1)	77.3 (70.0, 84.6)	46.7 (28.9, 64.6)	73.3 (70.4, 76.3)	*n* = 1,603; 74.6 (72.8, 76.4)	
	> 150%	13.9 (12.0, 15.8)	16.2 (13.5, 18.9)	5.5 (1.6, 9.5)	6.7 (0, 15.7)	14.4 (12.1, 16.7)	*n* = 302; 14.1 (12.6, 15.6)	
Education (ISCED)	Low	75.8 (73.4, 78.1)	75.2 (72.0, 78.4)	79.8 (72.9, 86.7)	83.9 (71.0, 96.8)	76.2 (73.4, 79.0)	*n* = 1,646; 76.0 (74.2, 77.8)	
Missing: 0	Intermediate	13.0 (11.1, 14.8)	15.2 (12.6, 17.9)	9.3 (4.3, 14.3)	9.7 (0, 20.1)	14.1 (11.8, 16.4)	*n* = 312; 13.4 (12.0, 14.8)	
	High	11.2 (9.4, 12.9)	9.5 (7.3, 11.7)	10.9 (5.5, 16.3)	6.6 (0, 15.3)	9.6 (7.7, 11.6)	*n* = 231; 10.5 (9.2, 11.8)	
Sex	Women	44.3 (41.6, 47.0)	60.3 (56.7, 63.9)	70.3 (62.4, 78.2)	83.9 (71.0, 96.8)	62.6 (59.4, 65.8)	*n* = 1,183; 51.7 (49.6, 53.8)	
Missing: 0								
Age group	18–34 years	19.8 (17.6, 22.0)	21.6 (10.1, 15.1)	18.8 (12.0, 25.6)	16.7 (3.3, 30.1)	21.1 (18.4, 23.8)	*n* = 457; 20.3 (18.6, 22.0)	
Missing: 0	35–54 years	33.3 (30.7, 35.9)	42.5 (38.8, 46.1)	30.5 (22.5, 38.5)	46.7 (28.9, 64.5)	40.9 (37.6, 44.2)	*n* = 812; 36.4 (34.4, 38.4)	
	55–74 years	31.8 (29.2, 34.3)	29.0 (25.6, 32.3)	39.1 (30.6, 47.6)	16.7 (3.3, 30.1)	30.0 (26.9, 33.1)	*n* = 710; 31.1 (29.1, 33.1)	
	≥ 75 years	15.1 (13.1, 17.1)	6.8 (4.9, 8.7)	11.7 (6.1, 17.3)	20.0 (5.7, 34.3)	8.0 (6.2, 9.8)	*n* = 210; 12.2 (10.8, 13.6)	
Place of residence	Urban	86.3 (84.4, 88.2)	89.5 (87.2, 91.8)	81.4 (74.7, 88.1)	83.9 (71.0, 96.8)	88.1 (85.9, 90.3)	*n* = 1,912; 87.0 (85.6, 88.4)	
Missing: 0								
Employment status	Employees	58.1 (55.4, 60.8)	65.8 (62.3, 69.3)	53.1 (44.5, 61.8)	54.8 (37.3, 72.3)	63.6 (60.4, 66.8)	*n* = 1,347; 60.3 (58.2, 62.4)	
Missing: 0	Unemployed	4,5 (3.4, 5.6)	3.7 (2.3, 5.1)	6.3 (2.1, 10.5)	12.9 (1.1, 24.7)	4.3 (3.0, 5.7)	*n* = 119; 4.4 (3.5, 5.3)	
	Non-employed	37.4 (34.8, 40.1)	30.5 (27.1, 33.9)	40.6 (32.1, 49.1)	32.3 (15.8, 48.8)	32.1 (29.0, 35.2)	*n* = 723, 35.3 (33.3, 37.3)	
Marital status	Married	56.9 (54.2, 59.6)	56.9 (53.2, 60.6)	54.3 (45.7, 62.9)	35.5 (18.7, 52.3)	55.8 (52.5, 59.1)	*n* = 1,076; 56.5 (54.4, 58.6)	
Missing: 8	Unmarried	21.6 (19.3, 23.9)	26.2 (22.9, 29.5)	14.7 (8.6, 37.3)	29.0 (13.0, 45.0)	24.6 (21.7, 27.5)	*n* = 580; 22.8 (21.0, 24.6)	
	Divorced	10.7 (9.0, 12.4)	10.9 (8.6, 13.2)	16.3 (9.9, 22.7)	12.9 (1.1, 25.0)	11.7 (9.6, 13.9)	*n* = 321; 11.1 (9.8, 12.4)	
	Widowed	10.8 (9.1, 12.5)	6.0 (4.2, 7.8)	14.7 (8.6, 37.3)	22.6 (7.9, 37.3)	7.9 (6.1, 9.7)	*n* = 204; 9.7 (8.4, 11.0)	
Living with partner	Yes	65.4 (62.8, 68.0)	68.4 (65.0, 71.8)	68.0 (60.0, 76.1)	48.4 (30.8, 66.0)	67.6 (65.6, 69.6)	*n* = 1,275; 66.3 (64.3, 68.3)	
Missing: 16								
Children < 18 years living in the household	Yes	21.3 (19.1, 23.5)	27.6 (24.3, 30.9)	22.7 (15.4, 30.0)	41.9 (24.5, 59.3)	27.3 (24.3, 30.3)	*n* = 482; 23.7 (21.9, 35.2)	
Missing: 1								
Obesity (BMI ≥ 30)	Yes	10.3 (8.6, 12.0)	18.0 (15.1, 20.9)	20.3 (13.2, 27.4)	59.4 (42.4, 76.4)	19.9 (17.2, 22.6)	*n* = 306; 14.2 (12.7, 15.7)	
Missing: 37								
Depressive symptoms (PHQ)	Yes	4.5 (3.4, 5.6)	8.0 (6.0, 10.0)	22.8 (15.5, 30.1)	35.5 (18.7, 52.3)	11.2 (9.1, 13.3)	*n* = 160; 7.2 (6.1, 8.3)	
Missing: 11								
Headache-specific physician consultation	Yes	-	46.4 (42.7, 50.1)	69.7 (61.5, 77.9)	90.0 (79.3, 100.0)	51.3 (47.9, 54.7)	-	-
Missing: 16								

*CI* Confidence interval, *ISCED* International Standard Classification of Education, *BMI* Body mass index, *PHQ* Patient Health Questionnaire, depressive subscale encompasses two items and has sum scores ranging from 0 to 6, scores ≥ 3 indicate depressive symptoms

### Association between SEP and headache prevalence

A binary logistic regression analysis was performed to investigate whether NEI and education predicted the probability of having headache. Sociodemographic, family and health-related characteristics, including depressive symptoms, were considered as control variables. Neither of the two SEP-variables was a significant predictor variable. A higher *OR* of having any type of headache was found for women, persons younger than 75 years, obese persons und those with depressive symptoms. All model coefficients and *OR* can be found in Table 2.

**Table 2:** Estimated effects of characteristics associated with the likelihood of headache: Binary logistic regression analysis (*N* = 2,189). Weighted random sample

Variable	All headaches				
	***B***	***SE***	***OR***	**95% ** ***CI***	***p*** ** value**
**Socioeconomic position (SEP)**					
Net equivalised income (NEI) (Ref.: NEI > 150%)					
NEI < 60%	-0.90	0.21	0.91	0.61, 1.38	.670
NEI 60–150%	-0.14	0.14	0.87	0.66, 1.16	.343
Education (ISCED) (Ref.: High education)					
Low education	0.19	0.16	1.21	0.88, 1.67	.236
Intermediate education	0.24	0.20	1.27	0.86, 1.86	.227
**Sociodemographic variables**					
Sex (Ref.: Men)	0.80	0.10	2.22	1.83, 2.69	< .001
Age (Ref.: ≥ 75 years)					
18–34 years	0.51	0.25	1.67	1.02, 2.74	.041
35–54 years	0.68	0.23	1.97	1.25, 3.12	.003
55–74 years	0.52	0.19	1.68	1.15, 2.46	.007
Place of residence (Ref.: Urban area)	-0.07	0.14	0.94	0.71, 1.24	.644
Employment status (Ref.: Employees)					
Unemployed	-0.14	0.14	0.87	0.66, 1.14	.299
Non-employed	-0.44	0.25	0.65	0.39, 1.06	.083
**Family variables**					
Marital status (Ref.: Married)					
Unmarried	0.32	0.18	1.37	0.97, 1.94	.073
Divorced	0.27	0.19	1.31	0.90, 1.91	.159
Widowed	-0.01	0.25	0.99	0.61, 1.61	.973
Living with partner (Ref: Living without partner)	-0.20	0.16	0.82	0.60, 1.13	.228
Children < 18 years living in the household (Ref.: No)	0.11	0.13	1.12	0.86, 1.46	.395
**Health-related variables**					
Obesity (BMI ≥ 30) (Ref.: No obesity)	0.79	0.14	2.20	1.68, 2.87	< .001
Depressive symptoms (PHQ) (Ref.: No depressive symptoms)	0.97	0.19	2.63	1.83, 3.80	< .001
**Constant**	-1.58	0.27	0.21		< .001
Model fitting: χ^2^ (df), *p*			178.87 (18), < .001		
*R* ^2^ (Nagelkerke`s)			.11		
Overall percentage of accuracy in classification			65.5		

*B* Unstandardized Beta weight, *SE* Standard error, *OR* Odds ratio, *CI* Confidence interval, *df* degree of freedom, *Ref*. Reference, *ISCED* International Standard Classification of Education, *BMI* Body mass index, *PHQ* Patient Health Questionnaire, depressive subscale encompasses two items and has sum scores ranging from 0 to 6, scores ≥ 3 indicate depressive symptoms

### Association between SEP and headache frequency

Ordinal logistic regressions were conducted to predict the effect of SEP on headache frequency (“LFEH”, “MFEH”, and “CH”) when additional factors are considered in a stepwise manner (Table 3). It was found that NEI, but not education, was significantly associated with headache frequency. Compared to participants with an NEI of more than 150% of median income, participants with an NEI of less than 60% and participants with an NEI of 60% to 150% of median income were more likely to report higher headache frequency (Model 1). This association was weakened but remained significant when sociodemographic and family variables (Model 2), and health-related variables were added (Model 3). Participants with an NEI of less than 60% of the median income were 5.21 times more likely to experience higher headache frequency (95%CI 2.03, 13.36). Participants with an NEI between 60 and 150% of the median income were 2.29 time more likely to experience higher headache frequency (95%CI 1.02, 5.11). Other significant predictors were widowhood, *p* = 0.004, obesity, *p* = 0.001 being classified with depressive symptoms, *p* < 0.001, and consultation with a physician due to headache, *p* < 0.001. All coefficients can be found in Table 3.

**Table 3:** Ordinal logistic regression for the association between socioeconomic position (SEP) and headache frequency (LFEH, MFEH, CH). (*N* = 891). Weighted random sample

Variable		Model 1			Model 2			Model 3		
		**B**	***SE***	**95% ** ***CI***	**B**	***SE***	**95% ** ***CI***	**B**	***SE***	**95% ** ***CI***
**Socioeconomic position (SEP)**										
Net equivalised income (NEI) (Ref.: NEI > 150%)										
NEI < 60%		1.90***	.40	1.11, 2.68	1.61***	.43	0.76, 2.46	1.65***	.48	0.71, 2.58
NEI 60–150%		.94**	.36	0.24, 1.64	.80*	.37	0.08, 1.52	.83*	.41	0.02, 1.63
Education (ISCED) (Ref.: High education)										
Low education		-.28	.31	-0.88, 0.33	-.38	.32	-1.01, 0.25	-.22	.39	-0.98, 0.53
Intermediate education		-.66	.40	-1.45, 0.13	-.63	.41	-1.43, 0.18	-.55	.49	-1.51, 0.41
**Sociodemographic variables**										
Sex (Ref.: Men)					.48*	.21	0.07, 0.88	.41	.23	0.03, 0.86
Age group (Ref.: 35–54 years)										
18–34 years					.21	,29	-0.35, 0.77	-.15	.32	-0.48, 0.77
55–74 years					.27	.29	-0.29, 0.83	.01	.31	-0.60, 0.62
≥ 75 years					.65	.52	-0.17, 1.47	.49	.45	-0.39, 1.37
Place of residence (Ref.: Living in an urban area)					.53*	.25	0.04, 1.02	.48	.26	-0.04, 1.00
Employment status (Ref.: Employees)										
Unemployed					.52	.40	-0.27, 1.31	.23	.43	-0.83, 0.28
Non-employed					-.24	.26	-0.74, 0.26	-.28	.28	-0.83. 0.28
**Family variables**										
Marital status (Ref.: Married)										
Unmarried					.27	.29	-0.29, 0.83	-.01	.37	-0.74, 0.71
Divorced					.21	.29	-0.35, 0.77	.67	.36	-0.03, 1.36
Widowed					.99*	.40	0.22, 1.77	1.23**	.40	0.40, 2.07
Living without partner (Ref.: Living with partner)					-.24	.29	-0.81, 0.34	-.37	.32	-0.98, 0.25
Children under 18 living in the household (Ref.: No)					.05	.25	-0.43, 0.53	-.23	.27	-0.76, 0.30
**Health-related variables**										
Obesity (BMI ≥ 30) (Ref.: No obesity)								.57*	.23	0.13, 1.02
Depressive symptoms (PHQ) (Ref.: No*)*								1.19***	.25	0.70, 1.68
Headache-specific physician consultation, (Ref.: No*)*								1.18***	.22	0.75, 1.61
Model fitting: χ^2^ (df)		31.94 (4) ***			62.18 (16) ***			132.83 (19) ***		
Goodness-of-Fit	(Pearson) χ^2^ (*df*)	17.24 (12)			756.84 (664)			923.35 (1065)		
	(Deviance) χ^2^ (*df*)	19.53 (12)			446.89 (664)			588.71 (1065)		
Pseudo-*R* ^2^ (Nagelkerke’s)		.032			.064			.22		
Test of Proportional Odds: χ^2^ (*df*)		8.74 (4)			25.18 (16)			24.97 (19)		

*B* slope estimate, *SE* standard error, *Ref.* Reference, *CI* Confidence interval, *df* degree of freedom, *ISCED* International Standard Classification of Education, *BMI* body mass index, *PHQ* Patient Health Questionnaire, depressive subscale encompasses two items and has sum scores ranging from 0 to 6, scores ≥ 3 indicate depressive symptoms

^*^ *p* < .05

^**^ *p* < .01

^***^ *p* < .001

## Discussion

The present German population-based study examined whether people are differently affected by headache disorders according to their SEP, measured by income and education when controlled for depressive symptoms, in addition to a broad set of further potentially confounding factors. For this purpose, we analysed headache prevalence, an aspect in which inconsistent findings prevail, and attack frequency, an aspect that has so far been limited to CH, while LFEH and MFEH have been largely neglected in discussions about the link between SEP and headache.

First, *headache prevalence* in a representative German sample was neither associated with income nor with education. Second, *attack frequency *was predicted by income. Our finding shows that individuals with low and medium income experience more headache attacks than those with high income. Differences were not explained by depressive symptoms or obesity. No differences were found between headache attacks and education. Furthermore, we demonstrated, that the association between income and headache frequency persisted even after controlling for headache-specific physician consultations.

Given the cross-sectional nature of the study design, we postulate a potential bidirectional relationship between income level and attack frequency. Since stress is the most commonly self-reported headache trigger [52], we assume higher stress levels in the low- and median- compared to those in the high-income group. For low-income individuals, high exposure to stress is well-documented. Lower income is associated with higher levels of allostatic load, which refers to the cumulative burden of chronic stress and life events [53]. In the median-income sector, various structural changes have occurred in Germany, including an increase in temporary employment contracts, part-time work, self-employment, low-wage rates, and jobs with a high risk of automation [54]. Empirical evidence indicates that approximately 60% of individuals with median income in Germany are concerned about their financial situation and retirement security [55]. Economic stress can directly increase susceptibility to headache attacks. Additionally, it may exacerbate headache attacks through sleep disturbances, which is the second most common trigger for headaches [52]. Furthermore, individuals experiencing economic stress are more likely to have difficulties with family and friends [56]. In addition, aerobic exercise and strength training, which are known to reduce headache attacks [57], are performed less frequently by those under economic stress [58]. Conversely, headache attacks can lead to financial burdens due to their impact on work. A study in the United States found that individuals with chronic headaches are less likely to be employed compared to those with low headache frequency [59].

Our results on *headache frequency* are largely consistent with previous studies but go beyond these in three aspects. First, they show that the social gradient is not limited to frequent [37, 60] and chronic headache [18, 37, 61], but applies to the entire spectrum from low frequency to chronic headache. Second, the higher frequency of attacks not only relates to people with low income [12, 37], but also includes people with a medium income. Third, we are the first to provide support that the association persists even when considering depressive symptoms as a potential confounding factor. The absence of an association between headache prevalence and SEP in our study corresponds to three-quarters of population-based previous [7, 9, 16, 19, 21, 22] and current European studies [11]. However, none of these analyses controlled for depressive symptoms. There is only one study on the association between SEP and prevalence that also considered depression, a Spanish population-based study of migraine [15]. Our finding that depressive symptoms independently predict headache prevalence is consistent with the results of that study. While in our study headache prevalence did not differ by SEP, in the Spanish study it did. In contrast to the European literature, studies from the United States consistently reported higher prevalences at lower SEP [10, 12, 20, 24, 62–64]. We attribute this discrepancy between U.S. American and European findings mainly to the greater income inequality in the US compared to European countries [65]. Our assumption that income-specific headache prevalence occurs primarily in countries with high income inequality is supported by findings from Brazil [17, 66], Turkey [13] and Spain [15].

The present study has several limitations: First, the results do not permit conclusions about specific types of headache, such as migraine and TTH. Second, the cross-sectional design is a important limitation, as it does not allow for conclusions regarding the directionality or development of the association between SEP and headache frequency. Longitudinal studies, however, support the Social Causation Hypothesis, which posits that low SEP is a risk factor for increased headache frequency [37]. It is also possible that individuals with a higher SEP receive better medical treatment. Additionally, according to the Social Selection Hypothesis, frequent headaches may negatively impact occupational performance and limit future employment opportunities, potentially leading to socioeconomic decline. Third, our sample included only a small number of individuals with CH, which may introduce bias. Finally, using a 6-month prevalence measure complicates comparisons with studies that used a 1-year prevalence.

The strengths of this study include the testing of different headache frequency groups (LFEH, MFEH and CH), as well as the inclusion of a wide range of possible confounding variables. Furthermore, the study cohort was randomly selected, population-based and representative of the German population aged ≥ 18 years.

Our results have several implications. To have greatest impact in reducing headache attacks, it is essential to target those affected in both low and middle-income groups. Universal prevention campaigns in the field of public health seem to be particularly suitable for this purpose. These campaigns should focus on both stress reduction and enhancement of coping skills. A combination of structural and behavioral preventive measures appears to be the most effective approach. Physicians should also be aware of the relationship between income and headache frequency in low- and middle-income patients. Moreover, this connection should be acknowledged by both stakeholders and practitioners.

## Conclusion

We found that while headache prevalence is not associated with socioeconomic position (SEP), headache frequency is. Among individuals with headaches, those with low- or middle-income are more likely to experience frequent headaches and are at a higher risk of developing chronic headaches. Physicians should be aware of the link between income and headache frequency, especially in low- and middle-income patients. This relationship should also be recognized by stakeholders and practitioners. Future research should investigate which protective factors contribute to a lower likelihood of high headache frequency among individuals with high income.

## Data Availability

The dataset generated and analyzed during this study is available from the corresponding author on reasonable request.

## Abbreviations

BMI: Body Mass Index
CH: Chronic Headache
CI: Confidence Interval
EH: Episodic Headache
GBD study: Global Burden of Disease study
GMV: Gray Matter Volume
ICHD-III: International Classification of Headache Disorders, third edition
ISCED-97: International Standard Classification of Education 97
LFEH: Low Frequency Episodic Headache
LL: Log-Likelihood
MFEH: Moderate Frequency Episodic Headache
NEI: Net Equivalised Household Income
OR: Odds Ratio
PHQ-4: Patient Health Questionnaire (4-item)
SEP: Socioeconomic Position
TTH: Tension-Type Headache
YLDs: Years Lived with Disability
